# What factors do make quality improvement work in primary health care? Experiences of maternal health quality improvement teams in three *Puskesmas* in Indonesia

**DOI:** 10.1371/journal.pone.0226804

**Published:** 2019-12-20

**Authors:** Ralalicia Limato, Patricia Tumbelaka, Rukhsana Ahmed, Sudirman Nasir, Din Syafruddin, Hermen Ormel, Meghan Bruce Kumar, Miriam Taegtmeyer, Maryse Kok

**Affiliations:** 1 Malaria and Vector Resistance Laboratory, Eijkman Institute for Molecular Biology, Jakarta, Indonesia; 2 Department of Clinical Sciences, Liverpool School of Tropical Medicine, Liverpool, United Kingdom; 3 School of Public Health, Hasanuddin University, Makassar, Indonesia; 4 KIT Royal Tropical Institute, Amsterdam, The Netherlands; 5 Department of International Public Health, Liverpool School of Tropical Medicine, Liverpool, United Kingdom; 6 Emory University, Rollins School of Public Health, Center for Humanitarian Emergencies, Atlanta, Georgia, United States of America; 7 Tropical and Infectious Disease Unit, Royal Liverpool University Hospital, Liverpool, United Kingdom; Tabriz University of Medical Sciences, ISLAMIC REPUBLIC OF IRAN

## Abstract

**Background:**

Indonesia has been shifting from ensuring access to health services towards improving service quality. Accreditation has been used as quality assurance (QA) mechanism, first in hospitals and subsequently in primary health care facilities, including *Puskesmas* (community health centres). QA provides measures of whether services meet quality targets, but quality improvement (QI) is needed to make change and achieve improvements. QI is a cyclical process with cycles of problem identification, solution testing and observation. We investigated the factors which influenced the process of QI based on experience of maternal health QI teams in three *Puskesmas* in Cianjur district, West Java province, Indonesia.

**Methods:**

Qualitative data were collected using 28 in-depth interviews at two points of time: pre- (April 2016) and post- QI intervention (April 2017), involving national, provincial, district and *Puskesmas* managers; and *Puskesmas* QI team members. Thematic analysis of transcripts was conducted.

**Results:**

We found four main factors contributed to the process of QI: 1) leadership, including awareness and attitude of leader(s) towards QI, involvement of leader(s) in the QI process and decision-making in budget allocation for QI; 2) staff enthusiasm and multidisciplinary collaboration; 3) a culture where QI is integrated in existing responsibilities; and 4) the ongoing *Puskesmas* accreditation process, which increased the value of QI to the organisation.

**Conclusion:**

Making QI a success in the decentralised Indonesian system requires action at four levels. At individual level, leadership attributes can create an internal quality environment and drive organisational cultural change. At team level, staff enthusiasm and collaboration can be triggered through engaging and tasking everyone in the QI process and having a shared vision of what quality should look like. At organisational level, QI should be integrated in planned activities, ensuring financial and human resources. Lastly, QI can be encouraged when it is implemented by the wider health system as part of national accreditation programmes.

## Introduction

Efforts to improve healthcare in Indonesia have shifted from focusing on access to healthcare to improving the quality of healthcare. This is reflected in the Indonesian Ministry of Health (MoH) national strategic plans for 2010–2014 and 2015–2019 [1–4]. Specifically, these plans refer to quality related to human resources for health, drugs and medical supplies, across several objectives and over time [2,3].

Despite improvements in the uptake of skilled birth attendance and facility delivery, maternal health issues still remain a public health concern in Indonesia. The country struggled to decrease the Maternal Mortality Rate (MMR) from 359/100,000 live births in 2012 to the Millennium Development Goal [5,6] of 102/100,000 live births in 2015, reaching just 305/100,000 [5]. Many causes of maternal death can be prevented or managed with evidence-based healthcare services that are available in Indonesia. Thus, these circumstances reflect that quality of the healthcare services might be sub-optimal or unreliable [7].

One MoH strategy to assess and ensure health service quality is hospital accreditation [8,9]. Since 2014, accreditation has been expanded towards primary care facilities [10]. The main provider of primary health services is the *Puskesmas* (community health centre), a category of public health facilities located at the sub-district level that deliver curative and preventive services and health promotion to the communities they serve [11].

However, Varkey [12] argues that solely focusing on quality assurance and quality control through accreditation will not significantly improve health outcomes. Continuous quality improvement (QI) approaches should be incorporated in the existing quality assurance methods to improve quality of care [13]. QI in health care is defined as a rigorous systematic process that focuses on continuous efforts and activities to achieve measurable improvement of health outcomes, system performance and professional development, with the ultimate goal to improve community health [12–15].

Most of the QI conducted in healthcare in low- and middle-income countries (LMICs) has focused on hospitals and higher-level health facilities. However, there are several studies available on QI aiming to improve maternal healthcare at the community level, particularly in Sub-Saharan Africa and Middle East. In Northern Ghana, QI teams at health post, health centre and hospital level were found to have a positive effect on key maternal and child health outcomes, including increased skilled delivery [16]. In South Africa, QI teams that included community health workers and their supervisors contributed to improved maternal health knowledge and feeding practices among women in the community [17]. Studies in Ethiopia [18] and Tanzania [19] report about community members being part of QI teams, resulting in increases in postnatal care attendance and general care seeking and healthy behaviours respectively. Iranian study [20,21] shows that involvement of pregnant women in the QI process give them positive pregnancy experience and is effective to improve the standard care of delivery. A few studies reported on facilitators of QI processes. In rural Tanzania, health workers mentioned new methods for monitoring progress and mentoring visits as motivating and supporting in improving maternal and newborn care [22]. An evaluation of the maternal and newborn health partnership in Ethiopia found that community-level QI members and health workers at health centre and district level reported that the district’s culture, in relation to supervision and learning, and leadership improved, and that their capacity in QI was built [18,23].

Between April 2016 and April 2017, the plan-do-study-act (PDSA) cycle method [24–26]–with plan, do, study and act stages in each cycle–was introduced in three *Puskesmas* in Cianjur district, West Java province. ‘Plan’ stage includes developing change idea, and determining the success indicators, how the data will be collected, who are the subjects, and the predicted outcome. ‘Do’ stage comprises performing and documenting the intervention, its results, and the challenges. ‘Study’ stage is when the QI teams analyse the results, compare results with the predicted outcome, and make reflections on the lessons learn. Finally, the ‘act’ stage is where QI teams adapt the change idea based on the analysed results and make preparation to plan the next cycle [25,26].

The intervention focused on the process to improve care delivery of maternal health by the village midwives. QI trainings were delivered, mainly, to the supervisors of the village midwives. Over the whole implementation period, the QI process was followed, to investigate the factors that influence (successes and failures of) the QI process in the three *Puskesmas*, which had never engaged with the concept of QI before.

In this paper we present factors that influenced the process of QI at *Puskesmas* level in Indonesia based on experience of maternal health QI teams in three *Puskesmas*. This study aimed to contribute to improving health service quality in the primary health care system in Indonesia.

## Materials and methods

### REACHOUT QI intervention

A QI intervention was implemented in the district health office (DHO) and three selected *Puskesmas* (hereafter referred to as *Puskesmas* A, B, and C), based on the recommendation from the DHO. The maternal health topic was chosen due to persisting maternal deaths in Cianjur. The *Puskesmas* have a similar geographical context, community culture and a high annual number of maternal deaths in comparison to the other *Puskesmas* in the district [27–29]. The QI intervention was initiated by the REACHOUT consortium, which focuses on improving the equity, effectiveness and efficiency of community-level health services in Indonesia and is working in five other countries.

The QI intervention activities included two training workshops and follow-up on the QI progress by the Indonesian REACHOUT team (three researchers those are the first, second and fourth author) who previously completed QI training delivered by a health development professional from LSTM.

The first training workshop was conducted in April 2016, attended by two DHO officers and three *Puskesmas* teams (comprised of the head of *Puskesmas* and the midwife coordinator). Over the course of this two-day workshop, the REACHOUT team introduced the basic QI concepts and how QI differs from quality assurance. The topic on how to implement a PDSA cycle was presented in a step-by-step participatory and interactive manner. After presentation of every step, the three *Puskesmas* teams sat together to progressively develop their own QI project and action plan. Readings related to QI implementation were provided prior to the workshop. The expected outputs were that the attendees would conduct a similar workshop in their own institutions for their staff, form a QI team and initiate the QI implementation process, as set out in the action plans.

Following the workshop, the REACHOUT team followed up on the progress of each institution. In the first three months, the progress of three of four institutions was slow. The DHO had not started the implementation due to budget constraints, and it would not start the QI until the 2017 financial year started. *Puskesmas* A only formed a QI team and *Puskesmas* C had developed an action plan but had not started the implementation of any of the activities noted in the plan. Meanwhile, the QI team of *Puskesmas* B had completed one PDSA cycle. All institutions conducted a dissemination meeting about QI intervention but did not fully replicate the workshop.

Therefore, we conducted a second workshop in August 2016, which was held in each *Puskesmas*. This time we included all *Puskesmas* staff and the established QI team as participants to introduce them with QI concept and PDSA cycle. After the workshop, each *Puskesmas* developed an action plan with specific activities for QI in maternal health, which are summarised in Table 1. The three *Puskesmas* had different levels of success in the outcomes of the QI intervention, where two measured improvements in different aspects of quality of maternal care and one did not.

**Table 1:** QI implementation of three *Puskesmas* in Cianjur district from April 2016 to April 2017.

	PUSKESMAS A	PUSKESMAS B		PUSKESMAS C
Profile in 2015				
Setting	Sub-urban	Sub-urban		Sub-urban
Population in the catchment area	Total: 80,649	Total: 70,152		Total: 55,159
	Male: 41,514	Male: 36,293		Male: 28,666
	Female: 38,955	Female: 33,859		Female: 26,493
Maternal health human resources:				
Midwife coordinator	1	1		1
*Puskesmas* midwife	5	8		3
Village midwife	16	18		13
Number of deliveries	1,586	1,240		1,157
Maternal deaths	5 (highest in the district)	3		2
QI team				
Size	3 people (Midwife coordinator & two *Puskesmas* midwives)	10 people (Head of *Puskesmas*, midwife coordinator & eight *Puskesmas* midwives)		7 people (Head of *Puskesmas*, midwife coordinator, three *Puskesmas* midwives & administration officer)
Year of QI team formation	April 2016	April 2016		August 2016
Frequency of meeting	Once a month	Once a month		- First four months: once a month - The next four months: one time
QI implementation				
Context	Across the three *Puskesmas*, the main issue was the persistent occurrence of maternal deaths in the previous three years. Therefore, the primary goal was to prevent maternal deaths through QI in maternal health services			
Problem identified	In 2015, one of 16 village midwives performed quality 10T ANC services. The most neglected service was performance of laboratory tests. Case management and health counselling were conducted but not according to the quality standard. This led to undetected risks and late management of dangers during pregnancy	In 2015, only 50% of the pregnant women utilised the KIA book (maternal and child health book) was believed to cause low knowledge and awareness of pregnant women about maternal health issues. In addition, less than 50% of the village midwives performed 10T ANC services		In 2015, two maternal death cases were caused by the non-compliance of one village midwife on the standard of delivery screening procedure, leading to high-risk cases not being referred from the village to the *Puskesmas*
	Note: 10T ANC services are ten activities that must be performed during antenatal care, those are 1) measure the weight and height of the pregnant women, 2) check the blood pressure, 3) measure the height of fundal uterus, 4) screen and give tetanus toxoid vaccination (minimum two times during pregnancy), 5) give iron tablet, 6) determine the nutrition status by measure the mid-upper arm circumference, 7) do laboratory test (haemoglobin, HIV, blood type and rhesus, malaria screening), 8) check the foetus’ presentation and heartbeat 9) case management, and 10) pregnancy health counselling			
Aim	To improve the quality of ANC services delivered by village midwives	To improve the knowledge of pregnant women about maternal health issues written in the KIA book	To improve the quality of ANC services delivered by village midwives	To improve the adherence of village midwives to the standard screening procedure for referral
PDSA cycle				
◾ Number of cycles	One (1)	Two (2)		One (1)
◾ Plan	Cycle 1	Cycle 1	Cycle 2	Cycle 1
- Purpose	To develop change idea	To develop change idea	To develop change idea	To develop change idea
- Intervention plan	1. Provision of haemoglobin digital test kit for the village midwives 2. Refresher training workshop on case management of high-risk pregnancy topics	1. Raise the number of pregnancy classes so that more pregnant women could attend the class 2. Deliver the topics in the pregnancy class using the KIA book so the pregnant women can easily re-read the information at home	1. Perform on-site supervision and direct feedback for village midwives in the village and during their *Puskesmas* shift 2. Conduct refresher training workshop on quality 10T ANC services and high-risk pregnancies (high blood pressure, anaemia, etc)	1. Transfer the village midwife who did not comply with the standard of delivery screening procedure from village to *Puskesmas* 2. Conduct refresher training workshop and feedback on all topics of high-risk pregnancy to all village midwives
- Subject	16 village midwives	207 pregnant women	18 village midwives	16 village midwives
- Success indicators	Improved percentage of village midwife who perform quality 10T ANC services from 6% (n = 1) to 50% (n = 8)	Increased knowledge of pregnant women regarding maternal health issues	Improved 10T ANC services performed by village midwives	Improved the village midwives’ compliance on delivery screening procedure, assessed by: The knowledge on high risk pregnancy must score at least 75/100 and the skills to do 21 steps of delivery screening procedure must score at a minimum of 90/100
◾ Do	1. The haemoglobin digital test kit was provided to the village midwives who did not have the kits in November 2016 2. Refresher training workshop was conducted for 16 village midwives in three different batches	1. An additional pregnancy class was started in nine of twelve villages from one pregnancy class to two classes 2. Pre- and post-test on the maternal health issues were conducted to assess the knowledge of pregnant women	1. QI team conducted an on-site observation to obtain data on the 10T ANC services performed by village midwives 2. A refresher training workshop on 10T ANC services was delivered by the QI team 3. On-site supportive supervision and feedback were performed by the supervisor of village midwives both in the village and at the *Puskesmas* 4. Endline data on 10T ANC services were collected	1. Transfer the one village midwife who did not comply from village to the *Puskesmas* and provide her with close supervision and technical guidance 2. A series of refresher training workshop (seven workshops) on high-risk pregnancy was conducted for all village midwives 3. Role plays were conducted on how to do the 21 steps of delivery screening procedure 4. Pre- and post- tests of knowledge on and skills to do delivery screening procedure were conducted
◾ Study	Data analysis revealed that 56% (n = 9) of village midwives performed quality 10T ANC services in 2016 (in comparison to 6% (n = 1) at baseline)	The mean score of pre- and post-test increased from 69.2/100 (n = 207) to 82.5/100 (n = 203)	- Baseline data revealed that only two of 18 village midwives performed 10T. Seven midwives complied with 9T and the other seven performed 8T. There were two midwives who only did 7T - After the QI, four village midwives performed 10T, and the number of midwives who performed 9T and 8T remained the same - There was improvement in compliance to do 10T ANC services by the village midwives - In this cycle, the QI team was able to identify the previous unidentified cases. They revealed that from January to June 2017 there were 224 cases of anaemia in pregnancy and 64 cases of protein-energy malnutrition (PEM) in pregnancy	- The mean score on knowledge of high-risk pregnancy increased from 39.3/100 (in the pre-test) to 80.75/100 (in the post-test) - The mean score of role-play skills on 21 steps of delivery screening procedure was 70/100 meanwhile the goal was minimum 90/100
◾ Act	1. To continue the provision of the haemoglobin test kit by *Puskesmas* 2. To perform the next PDSA cycle on supportive supervision	To continue the existed class and to add the number of pregnancy classes in the remaining three villages to be at least two classes per village	1. To continue the on-site supportive supervision 2. To conduct the cycle 3 based on the anaemia and PEM issues found in the cycle 2	1. To re-assess the roleplay intervention and develop an improved version 2. To perform the next PDSA cycle on supportive supervision

### Data collection and analysis

Qualitative data were collected at two time points: April 2016 (baseline) and April 2017 (endline) through in-depth interviews (IDIs) with purposively selected participants. During the baseline data collection, a total of eight IDIs were done, including participants from the DHO (maternal and child health staff) and *Puskesmas* (the head of *Puskesmas* and midwife coordinator) from the three *Puskesmas* who were going to be involved in the QI intervention. For endline data collection, we interviewed 20 respondents. National and provincial maternal health stakeholders and members of the *Puskesmas* QI teams were added as study participants. The number and characteristics of these participants and the selection criteria are presented in Table 2.

**Table 2:** In-depth interview participants.

Level	Type of participant	Selection criteria	Reason for selection	Data collected in April 2016	Data collected in April 2017
National	Ministry of Health officer	Worked at the managerial level	To obtain information on the QI perspectives and implementation at these level	-	1
	Indonesia Midwifery Association officer			-	1
Provincial	Provincial Health officer			-	1
	Indonesia Midwifery Association officer			-	1
District	DHO officer	Working experience >2 years in maternal health management and policy; attended the first QI workshop	To understand their perspectives about QI, past experiences with QI and later experience implementing QI following QI workshop (at the district and sub-district level)	2	2
Sub-district/ *Puskesmas*	Head of *Puskesmas*			3	5
	Midwife coordinator			3	3
	QI team member	Involved in the QI intervention		-	6
Total				8	20
Total baseline and endline				28	

The REACHOUT team developed various types of interview guides for IDIs according to participant characteristics that were translated into Indonesian and back translated for checking content-consistency (S1–S6 Files). The baseline instruments aimed to explore the perception of quality; QI and quality services delivered by village midwives; efforts, facilitators of and barriers to past efforts to improve quality; and the systems used to measure quality. The endline instruments sought to gain understanding on the changes in perception of quality and QI; the QI process including QI team formulation, how the QI team performed the PDSA cycle, the facilitators and barriers of QI process and sustaining the QI intervention; and the significant changes that occurred as a result of the QI process. In this paper, we present the results related to factors that influenced the QI process, and therefore most data come from the IDIs conducted during endline.

All IDIs were conducted in the Indonesian language by trained researchers from the REACHOUT team and the interview length was between 60 to 90 minutes. Prior to each interview, researchers explained the purpose and main topics of the interview. We asked and obtained written consent to perform and tape-record the interview from all participants. IDIs were conducted to assure that participants could be interviewed in their workplaces, allowing sensitive areas to be probed and avoiding issues of hierarchy affecting group discussion [30–32]. All interviews were digitally recorded, transcribed verbatim, and translated to English by independent translators. Research assistants checked the quality of the translations.

The coding process used open coding [33] in NVivo (v.11^™^) software, combined with a pre-defined framework of factors that could influence the process of QI as based on the topic guides. Emerging themes were discussed, and the coding was refined based on research team consensus. The coded transcripts were further analysed, “charted” and summarised in narratives for each theme and sub-theme.

### Ethical approval

The study protocol was approved by the Ethics Committee of Research in Health, Medical Faculty of Hasanuddin University, Makassar, Indonesia (No. 597 /H4.8.4.5.31/PP36-KOMETIK/2016).

## Results

### Baseline

The baseline interviews provided information related to perceptions about QI, efforts to perform QI and written QI policies. The eight interviewed participants had similar perceptions about QI (cyclic process which aims to improve quality) and their understanding about QI was focused on QA (a process to maintain the fulfilment of a desirable quality).

*“Regarding maternal health*, *we have specific SOP (Standard Operating Procedure) for the midwives*. *Everything we do (in health services) must be in line with the SOP*. *That is how we improve the quality services*.*”* (Midwife coordinator, *Puskesmas* C, baseline)

When asked about written policies related to QI, all participants referred to the head of *Puskesmas’* mandate letter to conduct activities required for *Puskesmas* accreditation.

In the discussions with the QI teams in three *Puskesmas* during the second workshop, it was revealed that despite SOPs being available for midwives, there were no process indicators and activities to assess whether the SOP is followed accurately. The quality assessment was based on the final outcome, measured by the number of maternal deaths. The factors that influence the QI process were never systematically assessed.

### Endline

From the endline interviews, we identified four main factors that influence the QI process from the perception of QI implementers in the *Puskesmas*: leadership, human resources, quality culture and *Puskesmas* accreditation.

#### Leadership

We found that leadership was perceived as the most prominent factor that influenced the QI process. Both leaders and QI team members identified three leadership elements to be instrumental: 1) awareness and attitude of the leaders towards QI; 2) direct involvement of the leaders in the QI process; and 3) decision making on the budget allocation for QI.

The data revealed that the awareness of leaders on the importance of and the benefits offered by QI was crucial. The head of *Puskesmas* A and B, through the QI workshop, obtained understanding on the importance of QI to support their ongoing programmes.

*“The QI workshop made us aware that it is important to have a strategic way of thinking and strategic planning*, *and then following it up with the interventions that we are capable of doing*.*”* (Head of *Puskesmas* B, female, endline)

Most QI team members confirmed this notion as reflected in the quote below.

*“The success of QI implementation depends on the leader*. *When the leader is passionate*, *it motivates me to do the process*.*”* (QI team *Puskesmas* B, female, endline)

A lack of awareness of the leader resulted in implementing the QI intervention (in *Puskesmas* C) solely because it was appointed by a higher level of authority, in this case the DHO.

*“We are involved in this QI process because we were appointed by the DHO*. *I don’t understand why our Puskesmas is always appointed if there is a new programme* … *At the beginning*, *we felt obliged to do this process*.*”* (Head of *Puskesmas* C, male, endline)

*“The head of Puskesmas does not have initiative to implement the QI programme*.*”* (QI team *Puskesmas* C, male, endline)

We also found that when the leader was directly involved in QI implementation, it led to the success of the QI. The close monitoring and follow up, especially when the QI team encountered problems, helped the QI team to implement the QI.

*“I helped the QI team to solve the problems they faced*, *especially in decision making on which issue must be prioritised*.*”* (Head of *Puskesmas* B, female, endline)

The statements from the head of *Puskesmas* was confirmed by some of the QI team members:

*“The main facilitator of the QI implementation is the support and involvement of the Puskesmas head*.*”* (QI team *Puskesmas* A, female, endline)

The third leadership related issue was the decision making on budget allocation for QI activities. QI implementation requires money, and in the *Puskesmas*, the leader is the one who has the mandate for budgeting. Therefore, the QI activities could only be implemented once the leader approved the budget needed for related activities. *Puskesmas* A showed an example of how the leader supported the QI activities through budget allocation for, among others, providing village midwives with Haemoglobin digital test sticks as part of the QI intervention and this was confirmed by the QI team members.

*“In terms of budget*, *this Puskesmas can manage its own funds and we just have to propose it to the planning team*. *In terms of budget*, *we got approval from the head of Puskesmas (to allocate some funds for QI activities)*.*”* (QI team *Puskesmas* A, female, endline)

#### Human resources

With regard to human resources, we found two factors that influenced the QI process. First, the enthusiasm of people in the institution; and second, the collaboration between all staff to conduct the QI.

As activities in the QI process required teamwork to conduct data collection and analysis and implementation of selected interventions, the enthusiasm of *Puskesmas* staff to do this was seen as a contributing factor by most QI team members. Both QI team members from *Puskesmas* A and B stated that the enthusiasm of their colleagues made them achieve their improvement goal.

*“One supporting factor that makes the quality improvement was achieved is the enthusiasm of my colleagues to do the field work*.*”* (QI team *Puskesmas* B, female, endline)

What made the team members enthusiastic was that they were involved in the whole QI process starting with conceiving of the idea of change. This made them feel to be an ‘owner’ throughout the process. This is reflected in the case study (Box 1) and the following quote.

*“We did the whole QI process as a team*. *For example*, *when we prioritised which problem that we want to initially intervene*, *besides the QI team*, *we also engaged village midwives*, *Head of Puskesmas and the nutrition QI team*.*”* (QI team *Puskesmas* A, female, endline)

**Box 1. Case study *Puskesmas* A**

In *Puskesmas* A, the QI principles were performed. This was visible from quality-oriented leadership, involving everyone in the QI process and joint decision-making. At start, the head of *Puskesmas* engaged all *Puskesmas* staff in the QI workshop to let them get exposed to the QI concept and process. This quality-oriented leadership was also shown by including everyone in the conception of the QI work. All *Puskesmas* staff from different programs and sections, namely maternal and child health, nutrition, health promotion, environmental health and the *Puskesmas* quality team which consisted of physicians, nurses, dentist and pharmacist were gathered to discuss maternal health issues. Everyone was given opportunity to apply their knowledge and ideas in the PDSA framework. First, they identified and prioritised which maternal health problem to tackle based on the urgency, cost and feasibility. This was followed by collective decision-making on the selected interventions and task division to perform interventions. Last, an action plan formatted in a Gantt-chart was produced as a guideline for the team to track their activities. All *Puskesmas* staff felt enthusiastic and embraced the QI, since they were confident on the quality of the data they gathered and contributed in the decision-making process. This was shown by, for example, a physician taking a role as a resource person in a refreshing workshop for village midwives.

The second human resource factor, which was related to the first, was the collective action of all actors in the *Puskesmas* to work together as a team. Oftentimes, each division in the *Puskesmas* works in isolation to achieve their team’s goal. The QI team in *Puskesmas* A and C experienced a different teamwork dynamic which influenced their QI process. QI team A felt that the teamwork between all staff in the *Puskesmas* tangibly improved the results as stated below.

*“The coordination*, *especially between nutrition team and maternal and child health team*, *is very good*. *This condition makes the problem identification faster than before*.*”* (QI team *Puskesmas* A, female, endline)

On the contrary, the QI team of *Puskesmas* C faced challenges of uncooperative behaviour of other *Puskesmas* staff who were not in the maternal and child health (MCH) division to execute the QI activities, mostly because of ‘ego-programming’ (finding one’s own division the most important).

*“Only few of my colleagues were responsive to do this process together*. *The rest was indifferent* … *The initial collaboration between the MCH team and other teams like the dentist*, *the general practitioner and other divisions (developed during the QI workshop) was discontinued*. *I think it was because we tackled the midwife problem*, *so the others felt that this QI implementation was solely the responsibility of the MCH team and not their responsibility*.*”* (QI team *Puskesmas* C, female, endline)

#### Quality culture

A quality culture can be defined as shared values, attitudes and behaviour of everybody in the organisation on working towards continuous QI. This was revealed by both leaders and QI team members as another factor that influenced the QI process. Both leaders and QI team members who treated QI as part of the daily job were more successful in the QI process in comparison to ones who did not. Most participants from *Puskesmas* C considered the QI intervention as an isolated program rather part of their daily work. This is reflected in these following statements.

*“The barrier to do QI is that we have limited time because we have another task to do*, *and this programme is colliding with other programmes*.*”* (QI team *Puskesmas* C, female, endline)

*“Because of the demand to do our daily routine activities*, *we do not use the QI checklist in every ANC service*.*”* (QI team *Puskesmas* C, female, endline)

This was in contrast with the situation as perceived by study participants in *Puskesmas* A and B.

*“The QI process doesn’t add to my workload because it is part of my daily job*.*”* (QI team *Puskesmas* A, female, endline)

*“The observations to the village midwives indeed required extra time*, *but I and my team were happy to do so*. *We enjoyed the process and the results*.*”* (QI team *Puskesmas* B, female, endline)

#### Puskesmas accreditation

During the one-year QI implementation, all three *Puskesmas* underwent accreditation. This process pushed the *Puskesmas* to develop several strategies to ensure services qualities, for example the development of written standard operating procedures for health providers, the formation of a quality team and the provision of a feedback scheme from the patients to the providers through the availability of patient satisfaction questionnaires. All informants at managerial positions and a few QI team members mentioned that accreditation and QI influenced each other.

*“When I noticed the big-three assessment points in accreditation*: *management*, *individual health and community health*, *all of them have the quality element*. *I can use the knowledge obtained from the QI workshop to improve the strategies to succeed accreditation… Prior to QI*, *we discussed some quality strategies*, *but they were like a splattered puzzle*. *However*, *after we learned about QI*, *we were able to put all the puzzle pieces together*.*”* (Head of *Puskesmas* A, female, endline)

The synergy between accreditation and QI was evident also when the accreditation assessor praised the maternal health section of *Puskesmas* B for their quality initiatives.

*“The QI process had synergies with the accreditation*. *Recently*, *the assessor team for accreditation expressed appreciation for the maternal health team of Puskesmas B as the best team for Puskesmas accreditation in Cianjur*. *I think their assessment was objective because they were from an independent body*.*”* (DHO officer, female, endline)

## Discussion

This study aimed to investigate factors that influenced the process of QI based on experience of maternal health QI teams in three *Puskesmas* which had never engaged with QI before. We found that the existence of leadership traits that support QI, individual enthusiasm, collaboration of everyone involved in the organisation, a teamwork approach, organisational quality culture and *Puskesmas* accreditation contributed to the success of the QI process in the *Puskesmas*. This became even clearer when assessing the differences between the QI interventions in *Puskesmas* A and B as compared to *Puskesmas* C. In *Puskesmas C*, the QI process lagged behind and less of the above described facilitators of QI were reported. As can be seen in Table 1, the implementation of the QI in this *Puskesmas* led to less positive, more mixed results with regard to knowledge and skills of community midwives as compared to *Puskesmas* A and B.

Following the findings of this study, the best approach to succeed in QI would be a multilevel approach. There are four levels where changes should occur: the level of the individual, team or group, organisation and the system where the organisation is embedded [34]. At the individual level, as found in studies elsewhere [35–39], leadership is seen as the foundation for successful QI. A leader is the one who can provide a clear vision and guidance of the organisational direction [40], create an internal quality management environment [40–42] and drive organisational cultural change [43]. One key element to ensure leader embarks in the QI process is quality literacy, which can be defined as competencies for QI–changing knowledge and values towards quality and competent behaviour to implement QI, that are obtained through quality education [44]. However, this study found that quality education can change knowledge but not necessarily leads to behaviour change. Three heads of *Puskesmas* (the organisations’ leader) attended the same QI workshop, but only two of them had a changed behaviour towards quality. One viewed QI as a burden to the existing workload. Therefore, leadership should be there at different levels in the organisation, in other words, leadership of (official) leader of the organisations might not be enough for a QI process to succeed.

Literature suggests that besides quality literacy, the availability of a quality policy and incentives for QI are needed to foster quality-oriented leadership. A quality policy underpins the planning and strategies for QI, since it creates an environment where quality of care can flourish, gives space to build capacity to improve quality [45], and sets frameworks for quality outcomes measurement and financial reforms towards QI [46]. Thus, it provides a leader a platform to perform QI, including in developing QI strategies and budget allocation. Another strategy to engage leader in QI is to provide incentives for QI through linking the compensated incentives to the quality metrics [47]. In this study, the differences in awareness and attitude between the leaders were related to personal traits rather than quality literacy, and the availability of quality policy and incentives.

Another leadership element that supports QI is the engagement of the leader in QI work. Other studies found that interaction between leader and staff when planning QI strategies was related to better quality outcomes, including patient health outcomes [48,49]. This study confirmed these findings as most QI team members in *Puskesmas* A and B perceived that the involvement of their leader in the QI process was crucial. The other facilitator of QI work in *Puskesmas* was found to be budget availability. Many primary health services operate on thin margins, and even though QI can reduce costs, at the introduction it often requires additional funds [50]. In the rather hierarchical health system of Indonesia, the budget allocation for *Puskesmas* operations depends on the decision of the leader–the head of *Puskesmas*. Therefore, the engagement of the leader in QI work is crucial in QI budget allocation. While maternal health is one of the priority programmes in Indonesia, and therefore the allocation of budget for QI in maternal health might be easier as compared to other programmes, leader and decision-makers need to be engaged in QI processes in general to ensure that necessary budget adjustments or relocations are made.

At the team or group level, two interrelated factors emerged as facilitators of QI implementation: enthusiasm of and collaboration between QI team members. There is no magic bullet to make people feel enthusiastic about QI work. However, it can be developed through setting short milestone achievements using the PDSA framework, in which the results, for example on intermediate outcomes related to maternal health, can provide information of what works and what does not work. This can make people feel the efforts pursuing a goal are worthwhile and wanting to do more [51]. Enthusiasm is also encouraged when the QI interventions meet the goal of the organisation [52], in the case of the *Puskesmas* who participated in this QI intervention, improvement of maternal health at community level. Besides, an organisational culture that supports personal contribution [53] and engagement of multidisciplinary actors in a team [22,54,55] provides an environment where improvement efforts can blossom.

At the organisation level, in this study, the existence of a quality culture emerged as a facilitator of the QI process. The changes in actions towards quality are rooted in the culture of the organisation [56]. The complexity of the quality culture lies in its development that requires changes towards quality at the two previous levels: the individual and the team level. A quality culture cannot be mandated but it has to be built through leadership and ownership [43,57–59]. Leadership sets the direction and articulates the vision of the organisation, and this trickles down through the organisation internal system. Quality-oriented leadership stimulates team members to adopt the same perspective and behaviour [59] and to implement quality-standard work performance [60]. At the same time, another crucial ingredient to create a quality culture is to give a sense of ownership to the staff through involving them in decision making [61], thereby stimulating leadership at different levels in the organisation. Findings in this study were in line with these notions as a quality culture was there in two *Puskesmas* that had quality-oriented leaders and enthusiastic staff, including midwives, who were involved in decision making.

At the system level, accreditation was mentioned as one of several strategies that facilitated QI. A study in Indonesia showed that *Puskesmas* accreditation had a significant positive correlation with quality services for patients, in relation to reliability of the providers and responsiveness to the patients’ needs [62]. Accreditation focuses on process and procedures, availability of written standards and compliance with standards, based on which recommendations are made by the surveyor [9,63,64]. It is administered by an independent body and based on voluntary participation of the accredited institution [65], although few can afford to not participate as it is a prerequisite for being included in the universal health coverage insurance scheme. However, to improve quality, it also takes internal reviews [65] and constantly seeking changes to ensure the standards and external review process are relevant [66]. Therefore, using a QI approach within a model that is compatible with accreditation leads to success, as also confirmed by our study participants. Further, to ensure the sustainability of QI, its embedment into the national and local policies and improvement strategies is paramount [15,55].

There are three limitations of this study. First, short data collection period (one year) limited the assessment of the end outcomes (number of maternal deaths). The participating *Puskesmas* only conducted at most two PDSA cycles over the one-year period in which the study was conducted. While a further assessment on the number of maternal deaths could not be conducted, intermediate outcomes were monitored, as presented in Table 1 under the ‘study’ stage of the PDSA cycle. Second, while all *Puskesmas* used anonymous scorecard filled by patients after they received maternal health services, quality measurement from health care users’ point of view was not presented in this paper. We have presented the QI process as performed by the organisation, including care delivery team and health care providers. Adding the health care users’ point of view on the process of QI in maternal health could have added valuable information, as presented in other studies [20,21,67]. Studies in Ethiopia and Tanzania, where community members were part of QI teams, have shown positive maternal health-related outcomes [18,19]. Third, the introduction of the intervention and the study that followed the process were both led by the same team. This could have influenced answers of study participants. However, given the fact that study participants reflected upon both barriers and facilitators in the QI process, we trust the validity of the study results.

## Conclusion

This study reviewed the factors influencing the process of QI in primary health care in Indonesia based on the experiences of maternal health QI teams in three *Puskesmas*. The process of QI in the decentralised Indonesian system is influenced by factors at four levels. At the individual level, leadership attributes create an internal quality environment and drive organisational cultural change. At the team level, staff enthusiasm and collaboration are built through engaging and tasking everyone in the QI process and having a shared vision of what quality should look like. Factors at the organisational level include integrating QI in the organisation structure and activities, securing financial resources and investing in human resources. Lastly, the existing accreditation system encouraged *Puskesmas* to implement and sustain QI approaches through external buy-in. QI should be implemented by the wider health system as part of national efforts to improve quality services at primary health care level.

## Supporting information

S1 File Interview guide QI baseline English. (PDF)

S2 File Interview guide QI baseline Indonesian. (PDF)

S3 File Interview guide QI team endline English. (PDF)

S4 File Interview guide QI team endline Indonesian. (PDF)

S5 File Interview guide QI stakeholders English. (PDF)

S6 File Interview guide QI stakeholders Indonesian. (PDF)
