# Supplementary material for: What factors do make quality improvement work in primary health care? Experiences of maternal health quality improvement teams in three Puskesmas in Indonesia
Source: PLoS One. 2019 Dec 20;14(12):e0226804. doi: 10.1371/journal.pone.0226804 (PMC6924663; doi:10.1371/journal.pone.0226804)
Supplement: S1 File — (PDF) [file pone.0226804.s001.pdf]

Interview guide Quality Improvement baseline English

1. In your view, what is the work that the village midwives (VMWs) should normally do? *(Interviewer: no more than 7 non-overlapping tasks)*
    a. Probe for specifics of curative, promotive, preventive activities, curative activities as well as non-health issues
    b. How is this communicated to them?
    c. How do you monitor/check if it's happening? (Probe: apart from checking the report)
    d. How much time each week should they spend on this?
    e. Do you have an estimate of how long is actually spent / how close this is to reality?

Fill in the below table with responses to questions in #1.

Task	Communication	Monitoring	Expected time or % of time		Actual time or % of time	
			Com	Mon	Com	Mon

2. What do you think of the quality of VMWs' work?
    a. Do they receive any feedback about this? How does it happen? From whom? How often?
3. How does the community judge the quality of VMWs' work?
    a. Do they have any opportunity to provide feedback? How? For example?
4. How do you know the work of VMWs is good quality?
    a. Does this agree with what the VMWs think? Why or why not?
    b. Is that important for you to know the quality of midwife work?
    c. Does district health office have any input into this?
    d. Does the national level have any input into this?
5. Does the health system measure this quality formally in any way?
    a. If yes, how? (list relevant tools, forums, etc.)
    b. Is anyone accountable for this quality through their performance objectives?
    c. Are there any informal measurements or reflections on this? For example?
6. How do you understand the term 'quality improvement'?
    a. How is this relevant to community health?
    b. How do you think communities see this? Supervisors? Providers themselves?
    c. Is there any written policy about quality improvement in your institution?
7. Can you give an example of how you and your colleagues have tried to improve the quality of community health work in the past? (formal or informal process)
    a. Probe for steps:
        i. How did you identify the issues/challenges?
        ii. How did you select the problem to work on from the list of challenges? How did you decide what was most important? What was feasible? Who decided?
        iii. How did you measure your improvement? Did it improve?

        iv. Who chose to solve the challenges?

8. Were there any barriers you experienced in doing QI in the past?
    a. Probe: Examples? Why do you think the challenge exists? How did you overcome it?
    b. Are there other barriers to your future QI work?
        i. How would you overcome those barriers?
        ii. What additional support would you need to overcome them? From whom?
9. What can supervisors do to support QI?
    a. How has this been done in the past? (Probe: time, budget allocation, training, staff, etc.)
    b. What can/should/must subordinates contribute?
    c. What other needs are there to be successful with future QI work?
10. Show showcard (next page) to respondents and help them to complete all section

Please take a look at the following table and indicate whether each listed item is present or not in your work and whether it is a facilitator or barrier to quality improvement work.

**Item**	**Present**			**Facilitator or barrier to high quality care**		**Explanation and comments** (e.g. understanding of the item, examples, etc.)
	Yes	No	Don't know	Facilitator	Barrier	
Supervision of CTC providers / budget for supervision						
Policy for quality of care at community level						
Guidelines on quality of care at comm. level						
Budget for QI						
Ongoing training of providers and/or supervisors						
Consumer/client/patient demands for specific services						
Availability of commodities for regular services						
Institutional support for QI and/or quality measurement						
Evidence availability for routine treatment approaches						
Availability of simple tools to measure quality						
"Culture of quality"						
Incentives for quality performance						

11. You just participated in QI workshop with REACHOUT. Have you been participated in the similar workshop in the past? Yes/No.
    a. Probe: What is the similarity of that workshop with REACHOUT workshop? What is the difference of that workshop with REACHOUT workshop?
12. Does the REACHOUT QI workshop beneficial for you? Yes/No. Why? Explain.
13. What are your suggestions relating to QI workshop REACHOUT had conducted?
14. After participating in REACHOUT QI workshop, do you have a plan to do QI in your institution? Yes/No. If yes, please explain. If no, why?
15. What is your goal of implementing the QI in your institution? How feasible is your goal? How do you want to measure it? When do you want to achieve that goal?
16. What activities will do you to achieve your goal?
    a. Probe: problem identification, problem prioritisation, problem analysis, building QI team, determines solution, implement solution, collecting data, analise (evaluate) data, revise solution (take action).
