# Supplementary material for: What factors do make quality improvement work in primary health care? Experiences of maternal health quality improvement teams in three Puskesmas in Indonesia
Source: PLoS One. 2019 Dec 20;14(12):e0226804. doi: 10.1371/journal.pone.0226804 (PMC6924663; doi:10.1371/journal.pone.0226804)
Supplement: S2 File — (PDF) [file pone.0226804.s002.pdf]

Interview guide Quality Improvement baseline Indonesian

1. Dalam pandangan Anda, apa saja tugas dan tanggung jawab yang biasanya dilakukan oleh bidan desa? (Pewawancara: tidak lebih dari 7 tugas yang tumpang tindih)
   a. Jajaki untuk tugas khusus dalam:
      - pelayanan promotif (promosi kesehatan),
      - pelayanan preventif (pencegahan penyakit),
      - pelayanan kuratif (pengobatan penyakit) atau manajemen kasus atau tindakan rutin,
      - hal – hal lain diluar masalah kesehatan?
   b. Bagaimana hal ini dikomunikasikan dengan mereka? Contohnya?
   c. Bagaimana Anda memonitor/mengecek apakah hal – hal tersebut dilakukan atau tidak? Contohnya? (Jajaki: pendekatan lain diluar laporan)
   d. Berapa banyak waktu yang bidan desa habiskan per minggu untuk melakukan hal ini?
   e. Apakah Anda bisa memperkirakan lamanya waktu yang dihabiskan untuk melakukan komunikasi dan monitoring dibandingkan dengan realita yang ada?

Mohon untuk mengisi tabel di bawah ini untuk menjawab pertanyaan no 1

**Tugas bidan desa**	**Bagaimana hal ini dikomunikasikan?**	**Bagaimana hal ini dimonitor?**	**Waktu yang diharapkan**		**Waktu sebenarnya**	
			**K**	**M**	**K**	**M**

2. Apa pendapat Anda tentang kualitas dari kinerja bidan desa?
   a. Apakah mereka mendapat umpan balik tentang hal ini? Kapan? Dari siapa? Seberapa sering?
3. Bagaimana masyarakat menilai kualitas dari pekerjaan bidan desa?
   a. Apakah mereka mempunyai kesempatan untuk memberikan umpan balik? Bagaimana? Atau Contohnya?
4. Bagaimana Anda mengetahui jika kualitas kinerja bidan desa baik?
   a. Apakah bidan desa mempunyai pendapat yang sama dengan Anda? Mengapa ya/mengapa tidak?
   b. Apakah penting bagi Anda untuk mengetahui kualitas kinerja bidan desa?
   c. Apakah ada masukan dari tingkat dinas kesehatan kabupaten tentang hal ini (kualitas kinerja bidan desa)?
   d. Apakah ada masukan dari tingkat nasional tentang hal ini (kualitas kinerja bidan desa)?
5. Apakah sistem kesehatan sudah mengukur kualitas ini secara formal?
   a. Jika ya, bagaimana? (minta partisipan menyebutkan instrument apa yang digunakan dan forum apa yang digunakan untuk proses ini)
   b. Apakah ada orang yang cukup kompeten untuk menilai kinerja bidan bidan desa secara objektif? Siapa/bagaimana Anda tahu jika orang itu cukup kompeten?
   c. Apakah ada penilaian tidak formal tentang hal ini? Jelaskan/contohnya?
6. Menurut Anda, apa yang dimaksud dengan menjamin kualitas *(quality assurance)*?
   Menurut Anda, apa yang dimaksud dengan meningkatkan kualitas *(quality improvement)*?

   a. Bagaimana peningkatan kualitas ini relevan dengan kesehatan masyarakat? Contohnya?
   b. Menurut Anda, apa pandangan masyarakat tentang peningkatan kualitas? Atasan? Bidan desa?
   c. Apakah ada kebijakan tertulis tenang peningkatan kualitas?
7. Dapatkan Anda memberikan contoh bagaimana Anda (atau rekan kerja Anda) melakukan peningkatan kualitas layanan kesehatan masyarakat dimasa lalu? (Baik secara formal maupun tidak formal).
   a. Jajaki semua langkah di bawah:
      i. Bagaimana Anda mengidentifikasikan masalah?
      ii. Bagaimana Anda memilih/memprioritaskan masalah dari sekian banyak masalah untuk Anda selesaikan? Bagaimana Anda memilih masalah yang paling penting untuk diselesaikan terlebih dahulu?
      iii. Apakah penyelesaian masalah tersebut memungkinkan untuk dilakukan?
      iv. Siapa yang menentukan/memilih intervensi penyelesaikan masalah?
8. Apakah ada hambatan yang Anda hadapi saat melakukan usaha peningkatan kualitas tersebut? Apa saja hambatan – hambatan yang Anda alami?
   a. Mengapa hambatan ini ada? Bagaimana Anda mengatasi hambatan tersebut?
   b. Apakah Anda merasa ada hambatan yang akan Anda alami saat melakukan usaha peningkatan kualitas di masa mendatang?
      i. Apa rencana Anda mengatasi hambatan – hambatan tersebut?
      ii. Dukungan apa yang Anda butuhkan untuk mengatasi hambatan – hambatan tersebut? Dari siapa?
9. Apa saja yang dapat dilakukan oleh atasan untuk mendukung usaha peningkatan kualitas?
   a. Bagaimana hal ini dilakukan dimasa lalu? Jajaki: waktu, alokasi dana, pelatihan, insentif, dll
   b. Kontribusi apa yang dapat/harus bawahan lakukan?
   c. Hal – hal lain apa lagi yang dibutuhkan agar usaha peningkatan kualitas dapat berhasil dimasa depan?
10. Tunjukkan tabel di bawah dan minta partisipan mengisinya

"Mohon Anda melihat tabel dibawah, mengisinya sesuai dengan keadaan yang Anda alami dan tentukan apakah keadaan tersebut menjadi faktor pendukung atau faktor penghambat usaha Anda melakukan peningkatan kualitas."

**Penjelasan**	**Ada tidaknya**			**Apakah ini menjadi**		**Penjelasan dan komentar** (contoh: pengertian tentang hal tersebut, contoh, dsb)
	**Ada**	**Tidak**	**Tidak tahu**	**Faktor pendukung**	**Faktor penghambat**	
Supervisi bidan desa						
Dana untuk supervisi						
Kebijakan (*policy*) tentang kualitas layanan kesehatan pada tingkat masyarakat						
Petunjuk (SOP) tentang kualitas layanan kesehatan pada tingkat masyarakat						
Dana untuk usaha meningkatkan layanan kesehatan masyarakat						
Pelatihan yang berkelanjutan untuk bidan desa						
Pelatihan berkelanjutan untuk atasan bidan desa						
Tuntutan pasien/klien untuk mendapatkan layanan spesifik (diluar yang mereka biasa dapatkan)						
Ketersediaan sarana (tempat, alat, bahan, obat) untuk layanan sehari – hari						
Dukungan dari institusi untuk upaya peningkatan kualitas kesehatan masyarakat						
Dukungan dari institusi untuk penilaian kualitas kerja bidan desa						
Ketersediaan sarana (tempat, alat, bahan, obat) untuk penatalaksanaan kasus rutin						
Ketersediaan instrumen sederhana untuk menilai kualitas *(checklist)*						
"Budaya mutu"						
Insentif/penghargaan jika melakukan pelayanan yang bermutu						

11. Anda sudah berpartisipasi dalam pelatihan QI oleh REACHOUT. Apakah Anda pernah berpartisipasi dalam pelatihan yang serupa sebelumnya? Ya/Tidak.
    a. Jajaki: Apakah kesamaan dengan pelatihan REACHOUT? Apakah perbedaannya?
12. Apakah pelatihan ini bermanfaat bagi Anda? Ya/Todak. Mengapa? Jelaskan.
13. Apa saran Anda sehubungan dengan pelatihan ini?
14. Setelah berpartisipasi dalam pelatihan ini, apakah Anda berencana untuk melaksanakan QI di institusi Anda? Jika tidak, mengapa?
15. Apa tujuan Anda mengimplementasi QI di institusi Anda? Apakah tujuan Anda dapat dicapai? Bagaimana Anda akan mengukurnya? Kapan Anda akan mencapai tujuan tersebut??
16. Kegiatan apa yang Anda akan lakukan untuk mencapai tujuan tersebut?
    a. Jajaki: identifikasi masalah, prioritisasi masalah, analisis masalah, membangun tim QI, menentukan solusi, mengimplementasi solusi, mengumpulkan data, menganalisis (evaluasi) data, merevisi solusi (mengambil tindakan selanjutnya).
