# Supplementary material for: What factors do make quality improvement work in primary health care? Experiences of maternal health quality improvement teams in three Puskesmas in Indonesia
Source: PLoS One. 2019 Dec 20;14(12):e0226804. doi: 10.1371/journal.pone.0226804 (PMC6924663; doi:10.1371/journal.pone.0226804)
Supplement: S3 File — (PDF) [file pone.0226804.s003.pdf]

Interview guide Quality Improvement team endline English

KNOWLEDGE AND PERCEPTIONS OF QI

1. What's your understanding of the term 'quality improvement' (QI)?
   a. How is this relevant to community health?
2. Overall, what has been your perception of the QI work you have been a part of over the last year?
   a. What worked well?
   b. What could have been better?
3. Why did you attend the QI team trainings? meetings?
   (*First ask this as an open-ended question and record spontaneous response. After that, please ask for each of the below areas (A-D). For all responses, probe for description/further understanding)*
   a. It was tied to a regular meeting
   b. Supervisor recommendation or decision
   c. Per diems provided
   d. Interest in the topic area
   e. Other?

QI TEAMWORK IN CYCLE 2 IN REVIEW

4. You and your colleagues on the QI team have tried to improve the quality of community health work. How did you identify the problems?
   a. Did you feel you had a voice in the process?
   b. Did you consult anyone outside of the QI team to determine this? (probe for: community health providers? Community members? Your supervisors?)
5. How did you select the problem to work on from the list of challenges?
   a. How did you decide what was most important?
   b. What was feasible?
   c. What was the decision process – who decided?
   d. Were there any other parties involved in this process?
6. How did you measure improvement?
   a. Probe for the tools used; existing monitoring tools or new?
   b. Why did you choose this approach?
   c. Did this approach success l to measure change?
7. Did you see an improvement or not yet?
   a. What helped this improvement to occur? (e.g. partnerships, new policies, leadership, etc.)
   b. Are there any barriers you experienced in doing this QI work that might have reduced or slowed its impact? If so, please describe.
8. What was the impact of QI on your workload?
   a. Specifically, what changed?
   b. Was that the same for your colleagues on the QI team?
   c. Did you find this acceptable?

QI TEAM FUTURE WORK / EMBEDDING

9. What problem does the QI team plan to work on next?
   a. When is your next meeting?
   b. How often do you plan to meet in the next 6 months?
10. What do you view as the main facilitators and barriers to your future QI work?
    a. How would you overcome those barriers?
       i. What additional support would you need to overcome them?

ii. Specifically:
1. What can the QI team itself do to support continuous QI?
2. What can supervisors do?
3. What can CTC providers do?
4. Anyone else?

11. On a scale of 1-5 with 5 being certain, what is the likelihood that the QI team's regular meetings will continue for at least one year? Why do you say that?
12. Finally, what is the most significant change you have experienced or observed because of the QI work? *(note this could be individual or institutional; including relationships, tools, protocols etc.)*
    a. Why is it important to you?
    b. How was the situation before REACHOUT and what is it like now?
    c. Do you hope it will change further? In what way?
