# Supplementary material for: What factors do make quality improvement work in primary health care? Experiences of maternal health quality improvement teams in three Puskesmas in Indonesia
Source: PLoS One. 2019 Dec 20;14(12):e0226804. doi: 10.1371/journal.pone.0226804 (PMC6924663; doi:10.1371/journal.pone.0226804)
Supplement: S4 File — (PDF) [file pone.0226804.s004.pdf]

Interview guide Quality Improvement team endline Indonesian

PENGETAHUAN DAN PERSEPSI TENTANG QI

1. Apakah pengertian Anda tentang ‘Quality Improvement’ (Peningkatan Kualitas)?
    a. Bagaimana hal ini relevan dengan kesehatan masyarakat?
2. Secara umum, apa persepsi/pendapat Anda tentang kegiatan ‘QI’ dimana Anda terlibat didalamnya dalam satu tahun terakhir (beberapa bulan terakhir)?
    a. Hal – hal apa saja yang sudah berhasil?
    b. Hal – hal apa saja yang belum berhasil atau perlu ditingkatkan?
3. Apa alasan Anda mengikuti pelatihan ‘QI’? pertemuan tim ‘QI’?
   Tanyakan pertanyaan terbuka dan minta jawaban spontan. Setelah itu, jajaki setiap area di bawah ini (A-D). Untuk semua respon, jajaki lebih jauh:
    a. Karena bersamaan dengan pertemuan rutin.
    b. Karena di wajibkan oleh atasan.
    c. Karena disediakan perdiem.
    d. Karena tertarik dengan ide dan kegiatan ‘QI’.
    e. Lainnya (sebutkan)

KEGIATAN TIM ‘QI’

4. Anda dan rekan kerja Anda sudah melakukan kegiatan QI untuk meningkatkan kualitas layanan masyarakat. Bagaimana cara Anda mengidentifikasi masalah?
    a. Apakah/bagaimana Anda merasa berperan dalam proses tersebut?
    b. Apakah Anda melakukan diskusi atau konsultasi dengan orang lain di luar tim QI untuk mengidentifikasi masalah tersebut? Jajaki: masyarakat, pimpinan, tenaga kesehatan?
5. Bagaimana Anda memprioritaskan satu masalah dari beberapa masalah yang Anda identifikasi?
    a. Bagaimana Anda memutuskan masalah mana yang paling penting untuk diselesaikan terlebih dahulu?
    b. Bagaimana Anda memutuskan masalah mana yang paling memungkinkan untuk diselesaikan terlebih dahulu?
    c. Bagaimana proses pemilihan masalah? Siapa yang memutuskan?
    d. Apakah ada pihak lain yang terlibat dalam proses ini? Jajaki: pihak desa, kecamatan.
6. Bagaimana Anda mengukur peningkatan?
    a. Jajaki: instrumen yang digunakan, instrumen baru atau yang sudah ada sebelumnya?
    b. Mengapa Anda memilih pendekatan ini (untuk mengukur peningkatan)?
    c. Menurut Anda, apakah pendekatan ini berhasil mengukur peningkatan? Ya/tidak. Mengapa?
7. Apakah Anda sudah melihat adanya peningkatan atau belum?
    a. Hal – hal apa yang dapat membantu peningkatan ini terjadi? (contohnya koordinasi, kebijakan baru, kepemimpinan, dll)
    b. Apakah ada hambatan yang Anda alami dalam melakukan kegiatan QI yang menyebabkan terlambatnya keluaran atau dampak yang Anda harapkan? Jelaskan!
8. Apa dampak dari kegiatan QI terhadap beban kerja Anda?
    a. Secara khusus, apa yang berubah?
    b. Apakah rekan kerja dalam tim QI merasakan hal yang sama?
    c. Apakah beban kerja ini masih bisa diterima?

<u>**KEGIATAN TIM ‘QI’ DIMASA MENDATANG**</u>

9. Apa permasalahan selanjutnya yang tim QI Anda rencanakan untuk diselesaikan?

   a. Kapan pertemuan berikut dilaksanakan?
   b. Seberapa sering pertemuan tim QI yang Anda rencanakan dalam 6 bulan kedepan?

10. Apa saja faktor – faktor pendukung kegiatan QI yang akan Anda lakukan dimasa mendatang?
    a. Bagaimana Anda mengatasi hambatan tersebut?
        i. Hal – hal/bantuan apa yang Anda butuhkan untuk mengatasi hambatan tersebut?
        ii. Khususnya:
            1. Apa yang dapat dilakukan oleh tim QI untuk mendukung keberlanjutan kegiatan QI?
            2. Apa yang dapat dilakukan oleh atasan Anda untuk mendukung keberlanjutan kegiatan QI?
            3. Apa yang dapat dilakukan oleh bidan desa untuk mendukung keberlanjutan kegiatan QI?
            4. Siapa lagi yang dapat mendukung keberlanjutan kegiatan QI? Dinkes?
11. Dalam skala 1 – 5 (skala 1 sangat tidak yakin dan skala 5 sangat yakin), seberapa yakin Anda bahwa tim QI akan melakukan pertemuan secara rutin dan melakukan kegiatan/program dengan menggunakan pendekatan QI untuk identifikasi masalah, prioritas masalah, dan implementasi intervensi dalam satu tahun kedepan? Mengapa?
12. Apakah perubahan yang paling signifikan yang Anda lihat atau rasakan yang disebabkan oleh adanya kegiatan QI di Puskesmas? *(perubahan bisa secara individu, insititusi termasuk instrumen, protokol, hubungan antar staf/atasan & bawahan, dll)*
    a. Mengapa Anda menyebutkan bahwa ini merupakan perubahan yang paling signifikan?
    b. Bagaimana keadaannya sebelum implementasi QI? Bagaimana keadaannya sesudah implementasi QI?
    c. Apakah Anda mengharapkan adanya perubahan yang lebih lagi? Dalam hal apa?
