# Supplementary material for: What factors do make quality improvement work in primary health care? Experiences of maternal health quality improvement teams in three Puskesmas in Indonesia
Source: PLoS One. 2019 Dec 20;14(12):e0226804. doi: 10.1371/journal.pone.0226804 (PMC6924663; doi:10.1371/journal.pone.0226804)
Supplement: S5 File — (PDF) [file pone.0226804.s005.pdf]

Interview guide Quality Improvement stakeholders English

1. First, I want to discuss about the services provided by village midwives.
   a. What is the type of services do they deliver?
   b. Is the community involved in the work of the village midwife? If yes, how?
   c. Who are the supervisors of the village midwives? How does the supervision system work in practice?
   d. What do you think of the quality of village midwives' work? Which aspects are of good quality? Why? Which aspects need to be improved?
   e. Do village midwives receive any feedback about this (good quality work and work that needs improvement)? How does it happen? (From whom/how often)
2. How do you think the community perceives the quality of village midwives' work?
   a. Do they have any opportunity to provide feedback, to say what goes well and what doesn't go well and needs improvement?
3. How do you think supervisors measure the quality of the work of village midwives?
   a. Is this in line with what the village midwives themselves think about measuring the quality of their work? Why or why not?
   b. Would it be important that village midwives and supervisors have the same idea about quality and how to measure it? Please explain why.
   c. Does the provincial/national level have any input into this?
4. Does the health system, at national/provincial level, formally measure this quality in any way?
   a. If yes, how? (list relevant tools, forums)
   b. Is anyone at higher level (national/provincial) accountable for this quality through their performance objectives?
   c. Are there any informal measurements or reflections on this? Any examples?
5. What do you think about the term 'quality improvement'?
   a. How is this relevant to community health? Any examples?
   b. How do you think communities see this? Supervisors? Providers themselves?
   c. Is there any written policy on quality improvement?
6. Can you give examples of how you and your colleagues at national/provincial level have tried to improve the quality of community health work in the past? (formal or informal process)
   a. Probe for steps:
      i. How did you identify the issues/challenges?
      ii. How did you select the problem to work on from the list of challenges?
      iii. How did you decide what was most important? What was feasible? Who decided?
      iv. How did you measure the quality improvement of this issue? Did it improve in the end? Please provide examples
7. Are there any barriers you experienced in doing QI in the past?
   a. Probe: Examples? Why do you think the challenge exists? How did you overcome it?
   b. Are there other barriers to your future QI work?
      i. How would you overcome those barriers?
      ii. What additional supports would you need to overcome them? From whom?
8. What can be done to support QI?
   a. How has this been done in the past? (Probe: time, budget allocation, training, staff, etc.)
   b. What can/should/must subordinates contribute?
   c. What are other requirements or conditions to be successful with future QI work?
9. Show showcard (next page) to respondents and help them to complete all section

Please take a look at the following table and indicate whether each listed item is present or not in your work and whether it is a facilitator or barrier to quality improvement work.

Item	Present			Facilitator or barrier to high quality care		Explanation and comments (e.g. understanding of the item, examples, etc.)
	Yes	No	Don't know	Facilitator	Barrier	
Supervision of CTC providers / budget for supervision						
Policy for quality of care at community level						
Guidelines on quality of care at comm. level						
Budget for QI						
Ongoing training of providers and/or supervisors						
Consumer/client/patient demands for specific services						
Availability of commodities for regular services						
Institutional support for QI and/or quality measurement						
Evidence availability for routine treatment approaches						
Availability of simple tools to measure quality						
"Culture of quality"						
Incentives for quality performance						
