# Supplementary material for: What factors do make quality improvement work in primary health care? Experiences of maternal health quality improvement teams in three Puskesmas in Indonesia
Source: PLoS One. 2019 Dec 20;14(12):e0226804. doi: 10.1371/journal.pone.0226804 (PMC6924663; doi:10.1371/journal.pone.0226804)
Supplement: S6 File — (PDF) [file pone.0226804.s006.pdf]

Interview guide Quality Improvement stakeholders Indonesian

1. Di awal wawancara ini kita akan berdiskusi tentang bidan desa
   a. Apa saja jenis layanan yang diberikan oleh bidan desa?
   b. Apakah masyarakat terlibat dalam pekerjaan bidan desa? Jika ya, bagaimana?
   c. Siapa supervisor atau pengawas bidan desa? Bagaimana sistem pengawasan ini dilakukan dalam praktek sehari – hari?
   d. Apa pendapat Anda tentang kualitas kerja bidan desa dalam kenyataan dilapangan? Aspek apa saja yang menurut Anda sudah "berkualitas? Mengapa? Aspek apa saja yang perlu ditingkatkan (belum berkualitas)? Mengapa?
   e. Apakah bidan desa menerima umpan balik tentang hal ini, baik tentang kualitas kerja yang sudah baik dan juga tentang aspek – aspek yang perlu ditingkatkan (kurang baik)? Bagaimana umpan balik diberikan? Dari siapa, seberapa sering, dalam forum apa, dalam bentuk apa?
2. Menurut pendapat Anda, apa pendapat masyarakat tentang kualitas kerja bidan desa?
   a. Apakah mereka (masyarakat) mempunyai kesempatan untuk memberikan umpan balik kepada bidan desa baik tentang hal-hal yang sudah baik dan juga tentang hal-hal yang kurang baik kepada bidan desa?
3. Menurut pendapat Anda, bagaimana supervisor atau atasan bidan desa menilai kualitas kerja bidan desa?
   a. Apakah pendekatan ini sesuai dengan apa yang bidan desa pikirkan tentang bagaimana seharusnya kualitas mereka dinilai? Mengapa ya? Mengapa tidak?
   b. Apakah penting bagi kedua pihak, yaitu bidan desa dan supervisor/atasan bidan desa untuk memiliki persepsi yang sama tentang kualitas bidan desa dan cara menilai kualitas tersebut? Jelaskan mengapa?
   c. Apakah stakeholder di tingkat nasional/propinsi mempunyai masukan untuk hal ini?
4. Apakah dalam system kesehatan di tingkat nasional/propinsi, kualitas bidan desa ini dinilai secara formal?
   a. Jika ya, bagaimana? (sebutkan instrument dan forum yang relevan)
   b. Apakah ada stakeholder di tingkat nasional/propinsi yang bertanggungjawab menilai kualitas kerja bidan desa ini secara objektif?
   c. Apakah ada pendekatan informal yang dilakukan untuk menilai kualitas bidan desa? Contohnya?
5. Apa pendapat Anda tentang istilah "Quality improvement" (QI) atau "peningkatan kualitas"?
   a. Bagaimana hal ini relevan dengan kesehatan masyarakat? Contohnya?
   b. Apa pendapat masyarakat tentang QI? Apa pendapat bidan desa tentang QI? Apa pendapat atasan bidan desa QI?
   c. Apakah ada kebijakan tertulis khusus tentang peningkatan kualitas?
6. Dapatkah Anda memberikan contoh bagaimana Anda ditingkat nasional melakukan usaha peningkatan kualitas di masa lalu, baik formal dan informal?
   a. Jajaki langkah-langkah:
      i. Bagaimana Anda mengidentifikasi masalah?
      ii. Bagaimana anda memilih suatu masalah yang akan di atasi terlebih dahulu dari sekian banyak masalah yang teridentifikasi?
      iii. Bagaimana Anda memutuskan masalah apa yang lebih penting, yang harus didahulukan intervensinya? Masalah mana yang lebih memungkinan untuk dilakukan intervensi? Siapa yang memilih intervensi tersebut?
      iv. Bagaimana Anda mengukur peningkatan kualitas sehubungan dengan masalah tersebut? Dalam kasus ini, apakah terjadi peningkatan kualitas? Berikan contoh!

7. Apakah ada tantangan yang Anda hadapi sehubungan dengan pelaksanaan kegiatan QI atau “peningkatan kualitas” dimasa lalu?
    a. Jajaki: Contohnya? Mengapa tantangan ini ada? Bagaimana Anda mengatasi tantangan tersebut?
    b. Apakah ada tantangan yang Anda prediksikan dalam melakukan “peningkatan kualitas” dimasa mendatang?
        i. Bagaimana Anda akan mengatasi tantangan ini?
        ii. Dukungan apa saja yang Anda perlukan untuk mengatasi tantangan tersebut? Dari siapa?
8. Apa saja yang bisa dilakukan untuk mendukung peningkatan kualitas?
    a. Bagaimana hal ini dilakukan di masa lalu? Jajaki: waktu, alokasi dana, pelatihan, staff, dsb
    b. Apa saja kontribusi dari bawahan?
    c. Hal – hal lain apa yang menjadi pra-syarat berhasilnya peningkatan kualitas dimasa mendatang?
9. Tunjukkan tabel di bawah dan minta responden untuk melengkapi tabel tersebut!

"Mohon Anda melihat tabel dibawah, mengisinya sesuai dengan keadaan yang Anda alami dan tentukan apakah keadaan tersebut menjadi faktor pendukung atau faktor penghambat usaha Anda melakukan peningkatan kualitas

**Penjelasan**	**Ada tidaknya**			**Apakah ini menjadi**		**Penjelasan dan komentar** (contoh: pengertian tentang hal tersebut, contoh, dsb)
	**Ada**	**Tidak**	**Tidak tahu**	**Faktor pendukung**	**Faktor penghambat**	
Supervisi bidan desa						
Dana untuk supervisi						
Kebijakan (*policy*) tentang kualitas layanan kesehatan pada tingkat masyarakat						
Petunjuk (SOP) tentang kualitas layanan kesehatan pada tingkat masyarakat						
Dana untuk usaha meningkatkan layanan kesehatan masyarakat						
Pelatihan yang berkelanjutan untuk bidan desa						
Pelatihan berkelanjutan untuk atasan bidan desa						
Tuntutan pasien/klien untuk mendapatkan layanan spesifik (diluar yang mereka biasa dapatkan)						
Ketersediaan sarana (tempat, alat, bahan, obat) untuk layanan sehari – hari						
Dukungan dari institusi untuk upaya peningkatan kualitas kesehatan masyarakat						
Dukungan dari institusi untuk penilaian kualitas kerja bidan desa						
Bukti bahwa bidan desa memberikan layanan kesehatan sesuai protokol						
Ketersediaan instrumen sederhana untuk menilai kualitas *(checklist)*						
"Budaya mutu"						
Insentif/penghargaan jika melakukan pelayanan yang bermutu						
